# A Potential Pathway for the Synthesis of Biomass-Based Polyamide Monomer 2,5-Bis(aminomethyl)furan from 2,5-Furandicarboxylic Acid

**DOI:** 10.3390/molecules30224336

**Published:** 2025-11-08

**Authors:** Cong Wang, Xin Li, Junqi Zhao, Bin Sun, Enquan Wang, Xuhong Mu, Xiaoxin Zhang

**Affiliations:** State Key Laboratory of Petroleum Molecular & Process Engineering, SINOPEC Research Institute of Petroleum Processing, Beijing 100083, China; wangcong.ripp@sinopec.com (C.W.);

**Keywords:** 2,5-furandicarboxylic acid, cyanation, 2,5-dicyanofuran, heterogeneous catalysis, hydrogenation, 2,5-bis(aminomethyl)furan

## Abstract

In this study, the transformation of 2,5-furandicarboxylic acid (FDCA) to 2,5-bis(aminomethyl)furan (BAMF) is proposed and investigated for the first time. Using FDCA as the substrate, the process involves two key steps: first, converting FDCA to 2,5-dicyanofuran (DCF) via carboxy-cyanation, followed by the heterogeneous catalytic hydrogenation of DCF to produce BAMF. For the carboxy-cyanation, two ammoniation routes were compared, including the molten ammoniated dehydration route and the moderate ammoniated dehydration route. The difference between the ammoniation of bio-based cyclic dicarboxylic acid and that of petroleum-based aliphatic dicarboxylic acid was discovered. A moderate ammoniated dehydration route that is more suitable for bio-based cyclic dicarboxylic acid has been developed. SOCl_2_ was found to effectively activate the stable carboxyl group and act as a dehydrating agent, facilitating the dehydration of the intermediate 2,5-furandicarboxamide (FDAM) to DCF with higher efficiency. For the hydrogenation reaction of DCF, Raney Co exhibited excellent catalytic performance, achieving a 94.5% yield of BAMF from DCF. Based on industrial practice, this research represents the first exploration of the pathway from bio-based FDCA to BAMF, which opens a new line for the sustainable production of bio-based diamines.

## 1. Introduction

Furan-based derivatives such as 5-hydroxymethylfurfural (HMF) and 2,5-furandicarboxylic acid (FDCA) are important platform compounds. FDCA was rated by the U.S. Department of Energy as one of the 12 most promising bio-based platform compounds [1]. And it is a dominant substitution of terephthalic acid in the production of polyester. Reports from the literature indicate that 5-HMF is unstable during storage and a rehydration of HMF has often been observed, or insoluble humins have been formed by polymerization [2,3,4]. Galkin et al. have pointed out the instability of HMF at an ambient temperature environment [5]. The instability of HMF has limited its large-scale application. Compared with HMF, FDCA is a more stable bio-based platform compound, which is prepared by a further oxidation of HMF or a direct conversion of biomass [6,7]. FDCA contains two more stable carboxyl groups, which are more chemically stable than the hydroxyl and aldehyde groups of HMF. FDCA is now widely used in the synthesis of bio-based polyesters and polyamides [8,9]. Converting HMF into the more stable FDCA may be a valuable alternative solution for its instability issues. The more stable FDCA compound will be a more promising choice for further deep processing of biomass platform compounds. With abundant C-O single bonds and C=O double bonds, the transformation of bio-based FDCA to prepare nitrogen-containing chemicals would entail higher economic value.

**Figure 1:**
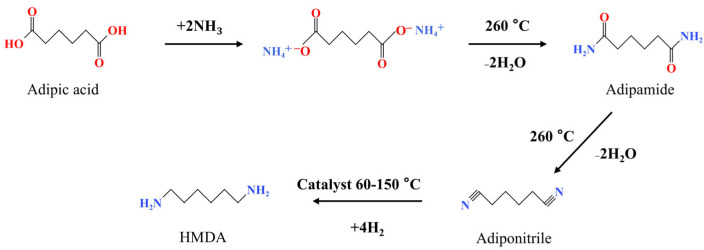
The synthesis of hexanediamine from adipic acid via molten ammoniated dehydration.

Bio-based amine compounds are widely used and have recently been studied intensively [10,11]. 2,5-Bis(aminomethyl)furan (BAMF), a furan-based diamine with an aromatic heterocyclic structure, plays an important role in the synthesis of polyamides and pharmaceuticals [12,13]. BAMF is a stable bio-based diamine with an unchanged furan ring (furan core) and two symmetrical aminomethyl groups. As a furan-based monomer, BAMF improves the heat resistance, mechanical properties and barrier properties of polymeric materials compared to chain diamines, due to the special natural barrier of motion and the stiffness of the furan ring [8,9,14]. This is expected to endow more functionality and added-value upon the polymeric materials. In addition, the monomer hexamethylenediamine (HMDA) of nylon 66 can also be synthesized by the hydrodeoxygenation of BAMF [15]. Current reports on the preparation of BAMF or 2,5-Bis-(aminomethyl)tetrahydrofuran (BATF) include the reductive amination of HMF, the reductive amination of HMFA and the amination of various derivatives of HMF [16,17]. All of these are still laboratory studies and have not yet been industrialized. The preparation of BAMF using FDCA has not been reported. Compared to HMF, FDCA is more stable and easier to store and transport, which make it more suitable as a raw material to manufacture bio-based BAMF. The preparation of BAMF using FDCA would be an interesting study and it is expected to achieve the large-scale manufacture of BAMF for polyamide materials. For the preparation of primary amine compounds by the amination of oxygen-containing functional groups, the reductive amination of carbonyl and hydroxyl functional groups is a commonly used and effective method [16,18,19,20,21]. The carbonyl functional group reacts with NH_3_ to produce imine, which is then hydrogenated to obtain primary amine [22]. And for the hydroxyl functional group, a carbonyl group is generated via dehydrogenation, which then goes further via the route of reductive amination of the carbonyl group [23]. The combination of these two reaction pathways contribute to HMF reductive amination for the preparation of BAMF [16,24,25]. However, the direct reductive amination of carboxylic groups has rarely been studied. Coeck et al. [26] first reported on the one-pot reductive amination of carboxylic acids to prepare primary amines, using RuWO*x*/MgAl_2_O_4_ as a catalyst and most monocarboxylic acids as reaction substrates, for which the yield of primary amines was high. However, it was very difficult to achieve the amination of dicarboxylic acids using this method, and the primary products were cyclization products, such as lactams or cyclic secondary amines. The highest yield of 30% was achieved in decamethylene diamine from the reductive amination of decanedioic acid, and yield of 0% was obtained for hexamethylenediamine (HMDA) from the reductive amination of adipic acid. Therefore, reduction amination is not considered a suitable method for binary carboxylic acids to prepare primary amines. To the best of our knowledge, the only industrialized example of diamine preparation from binary acid is the preparation of HMDA by ammoniation and the hydrogenation of adipic acid or adipic acid ester [27,28,29]. As shown in Figure 1, adipic acid reacts with ammonia to form the ammonium salt, which dehydrates to form adipic diamide, and then diamide dehydrates to form the adipic dinitrile, followed by hydrogenation to form HMDA. The dehydration is a heat-absorbing process that requires high temperatures, and the dehydration process temperature is as high as 260 °C [29]. This molten ammoniated dehydration pathway provides some ideas for the present work. In this work, a similar route was used in the previous exploration; however, a large gap was found between the thermal stability of the bio-based FDCA and the aliphatic adipic acid. Some related attempts were reported in this work, in which the FDCA was reacted with ammonia to form an ammonium salt, and it was found to be very difficult to produce an amide in a further dehydration. And the amide would be even more difficult to dehydrate in the following section. Dehydration requires high reaction temperatures, which contradicts the poor thermal stability of bio-based adipic acid FDCA. Therefore, the route of the ammoniation of aliphatic adipic acid is not suitable for bio-based FDCA. The route that is suitable for the preparation of diamines from bio-based diacids needs further thinking and exploration.

To convert the dicarboxylic group of FDCA to nitrogen-containing groups, it is necessary to activate the carboxyl group first. The use of electrophilic acid chlorides in aramid fiber gives us inspiration. Compared to the polymerization of aliphatic dicarboxylic acids and diamines, the preparation of aramid fiber from the reaction of terephthalic acid and terephthalamine is more difficult due to the influence of the benzene ring. Therefore, industrial aramids are prepared through the reaction of dichloride with diamines [30]. Industrially, carboxylic groups of terephthalic acid are replaced with a more reactive acyl chloride group through the use of thionyl chloride. And the product terephthaloyl dichloride could react more easily with diamines for the polymerization to prepare aramid fiber [31,32].

**Scheme 1:**
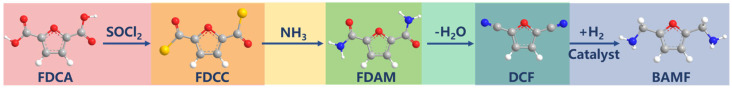
The synthesis pathway to BAMF from bio-based FDCA.

In this work, FDCA was first converted to active acyl chloride by an acylation reaction and amide was prepared by ammoniation, and then 2,5-dicyanofuran (DCF) was prepared by amide dehydration (Scheme 1). This innovative route avoided the contradiction that high temperature was needed for the dehydration of FDCA ammonium salt but high temperature gave rise to strong decarboxylation. The final step in the preparation of diamines by hydrogenation of DCF is also a critical step, as shown in Scheme 1. Transition metal catalysts were often used for the heterogeneous catalytic hydrogenation of nitriles. And the order of selectivity for primary amines was Co > Ni ≥ Rh ≥ Ru > Cu ≥ Pt ≥ Pd [33]. We have probed the reaction of DCF hydrogenation using commercial Raney Co and Raney Ni to reveal the reaction mechanism.

This study developed the efficient synthesis of bio-based polyamide monomer BAMF as the first reported example through a highly selective activation of C-O/C=O bonds in FDCA. The investigation provides a systematic elucidation of both the conversion pathway and the catalytic mechanism governing the transformation from FDCA to BAMF. These discoveries not only address a fundamental gap in understanding the rational preparation of bio-based diamine monomers from FDCA, but also demonstrate a transformative pathway to enhance the economic viability of biomass platform compounds. Beyond these contributions, the research establishes novel synthetic strategies for engineering furan derivatives, thereby laying robust foundations for the scalable conversion and industrial exploitation of biomass resources.

## 2. Results and Discussion

### 2.1. The Molten Ammoniated Dehydration of 2,5-Furandicarboxylic Acid

**Figure 2:**
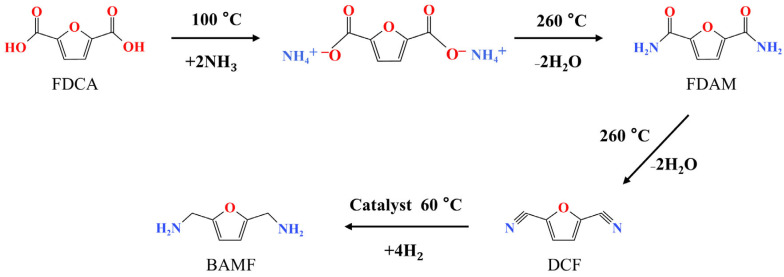
The route of molten ammoniated dehydration of FDCA (a conversion of FDCA to DCF through ammonium salt-amide-nitrile).

In the initial attempt, the preparation of 2,5-dicyanofuran from 2,5-furandicarboxylic was conducted by a molten ammoniated dehydration route as Figure 2 presented. Through the route of molten ammoniated dehydration, a small amount of the product DCF was detected. Figure S2 shows the HPLC chromatogram for the products collected in the separator, which presents the peak of product DCF and a larger peak of unconverted FDAM. The calculated yield of 2,5-dicyanofuran (DCF) was less than 1%.

This study found that the molten ammoniated dehydration route of adipic acid is not suitable for the preparation of 2,5-dicyanofuran from 2,5-furandicarboxylic acid. The carboxyl group is not a simple summation of the carbonyl and hydroxyl groups. And the carbonyl group in the carboxyl group becomes very inactive under the influence of the hydroxyl group [26]. The carboxylic hydroxyl hydrogen dissociates more readily than the alcoholic hydroxyl hydrogen and displays acidity. In the anion of the carboxylate, bond averaging occurs due to the off-domain action of the electrons. Therefore, a large amount of energy is required to break the C-O single bond of the ammonium salt produced from the carboxylic acid during the reaction, and carboxylic acids are usually converted into esters for further reaction [16,26]. Moreover, the furan ring is similar to the benzene ring and is known as (pseudo) aromatic furan, and 2,5-furandicarboxylic acid is not as active as adipic acid [14]. That is to say that the dehydration of ammonium 2,5-furandicarboxylic acid for the preparation of 2,5-furandicarboxylic acid in the high-temperature molten route requires a higher temperature, higher than that of adipic acid. And since 2,5-furandicarboxylic acid has a higher boiling point (419.2 °C ± 30.0 °C), much higher than that of adipic acid (338 °C), a higher temperature is also required to achieve melting of the reaction substrate FDCA. However, the bio-based 2,5-furandicarboxylic acid is thermally unstable above 200 °C. Figure S3 shows that the decomposition of FDCA occurs in N_2_ atmosphere at around 248 °C, and the decomposition products are mainly CO_2_ and H_2_O. It has also been reported that FDCA decarboxylates at around 195 °C [34]. In this study, yellowing of the solution in the reactor occurred before the melting temperature of FDCA was reached (Figure S4). And as the temperature of the reaction increased, the coking became more severe, and the by-products increased and were difficult to separate. Therefore, the high temperature required for ammonium salt dehydration contradicts the thermal stability of FDCA, which is the reason why biomass-based diacids cannot follow the route of molten ammoniated dehydration of fatty diacids. For the cyanation of biomass-based diacids with furan rings, like FDCA, reaction processes with milder reaction conditions need to be developed. Considering that acyl chlorides are more reactive than carboxyl groups, the present work proposes the FDCA-acyl chloride-amide-nitrile reaction pathway.

### 2.2. The Conversion of 2,5-Furandicarboxylic Acid to 2,5-Dicyanofuran by Acyl Chloride-Amide-Nitrile

**Figure 3:**
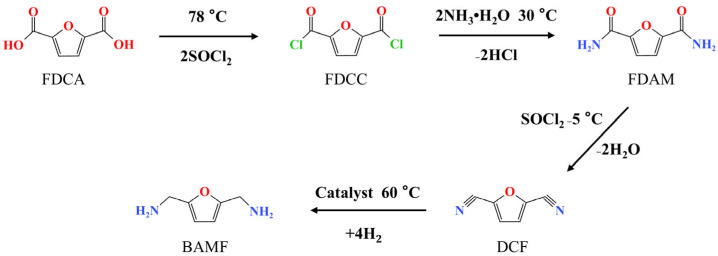
The route of moderate ammoniated dehydration of 2,5-furandicarboxylic acid (a conversion of 2,5-furandicarboxylic acid to 2,5-dicyanofuran by acyl chloride-amide-nitrile).

The reaction route of the FDCA-acyl chloride-amide-nitrile reaction path is shown in Figure 3. For the reaction of FDCA with SOCl_2_ to form dichloride, the yield of diacyl chloride could reach more than 99% (the molecular structure of FDCC is shown in Figures S8 and S9). In the reaction of diacyl chloride with ammonia, the yield of diamide was more than 99% (the molecular structure of FDAM is shown in Figures S6 and S7). The crucial reaction step lay in the dehydration of diamide to prepare dinitrile, which was fraught with difficulty. The results of the dehydration of diamide are shown in Table 1. The reaction was first conducted in a reflux process under 78 °C; the product can be seen in Figure S5. Under a high dehydration temperature, tar-like by-products were produced, and almost no DCF was detected in the product liquid. The black tar substance may come from DMF under a high temperature. DMF played a key role in promoting the dehydration of FDAM, and FDAM did not undergo the dehydration reaction when DMF was not added (Table 1 entry 3). When the reaction temperature was lowered to −5 °C, the dehydration effect was better than that of the high-temperature reflux, as shown in Table 1 entries 4–16. The ratios of DMF and SOCl_2_ additions were studied (Table 1, entries 4–8), and it was found that the yield of DCF could reach up to 64.0% when the stoichiometric ratios of SOCl_2_/FDAM and DMF/FDAM were 4.25 and 43, respectively (Table 1, entry 7). In order to further increase the DCF yield, the reaction time was optimized (Table 1, entry 7 and entries 9–16), which did not have a significant effect on the increase in DCF yield. As there was an equilibrium limit for the dehydration of FDAM in the SOCl_2_ and DMF system, the highest yield could be achieved by reacting for 7 h.

### 2.3. The Hydrogenation of DCF to BAMF

A catalytic process was applied to prepare BAMF by the hydrogenation of DCF on heterogeneous catalysts. In order to improve the selectivity of BAMF and analyze the mechanism of the reaction path, the reaction parameters including type of active metals, reaction solvent, reaction temperature, reaction time, and catalyst dosage have been studied and optimized.

**Figure 4:**
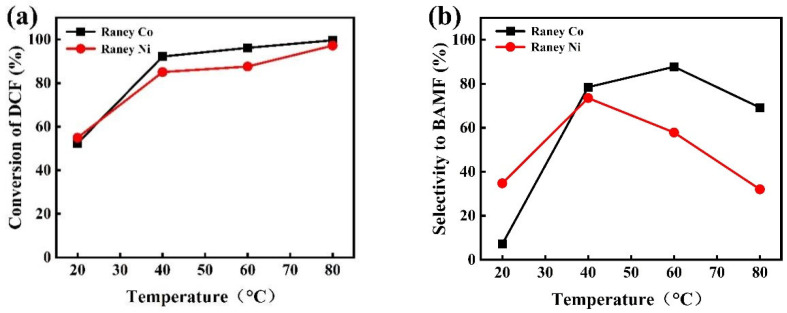
Comparison of hydrogenation effect of DCF under Raney Co and Raney Ni: (**a**) conversion of DCF; (**b**) selectivity to BAMF. (Reaction conditions: 0.2 g DCF, 2 MPa H_2_, 2 h, 0.1 g Catalyst, 0.05 g NaOH, 10 mL EtOH).

It is necessary to screen a suitable catalyst for the hydrogenation of DCF. Raney-type catalysts have a wide range of applications in the conversion of bio-oils [35] and the hydrogenation of glucose to sorbitol [36,37], as well as in nitrile hydrogenation processes, such as the preparation of n-butylamine by highly selective hydrogenation from butyronitrile [38] and the preparation of hexanediamine from adiponitrile [39]. Raney Co and Raney Ni were studied in this work for comparing their catalytic performance on DCF hydrogenation. As shown in Figure 4a, the conversion of DCF gradually increased with temperature increasing from 20 °C to 80 °C both with Raney Co and Raney Ni. The conversion with Raney Co was higher overall than that with Raney Ni. For the selectivity of BAMF, both Raney Co and Raney Ni showed a volcano curve with increasing reaction temperature (Figure 4b). The selectivity of Raney Co was also better than that of Raney Ni and achieved 87.7% at 60 °C (Table S2, entry 3).

Factors of catalysts affecting the reaction performance of active metals usually include metal intrinsic activity, particle size, the surface morphology of active metal and so on. In this study, Raney Co and Raney Ni both have a particle size of 50 μm (commercial purchase). Therefore, the particle size is not considered to be the reason of the difference in activity and selectivity between these two catalysts. The active metals used in nitrile hydrogenation for amine preparation are mainly transition metals. And the selectivity of these metals is in the descending order of Co > Ni > Ru > Rh > Cu > Pt > Pd [33]. The metal intrinsic activity between Raney Co and Raney Ni perhaps lead to the different catalytic performances when converting DCF into BAMF.

The textural properties of Raney Ni and Co catalysts were systematically characterized by N_2_ adsorption–desorption analysis. Raney Ni displayed a distinct type IV isotherm with a well-defined H3-type hysteresis loop in the relative pressure range of 0.4–1.0 (Figure S10), unequivocally confirming its mesoporous architecture. Notably, the sharp uptake at high P/P_0_ regions reflects the abundance of mesopores with broader pore size distribution, substantiated by the significantly larger specific surface area and pore volume compared to Raney Co (Table S1). In contrast, Raney Co exhibited a more gradual isotherm slope at elevated pressures, indicative of a pore structure dominated by micro- and macroporous features rather than mesopores. These substantial textural differences directly correlate with the catalysts’ performance: Raney Ni’s superior surface area and developed mesoporosity provide substantially more accessible active sites, explaining its enhanced hydrogenation activity that leads to over-hydrogenation in DCF reduction when exceeding optimal temperatures.

**Figure 5:**
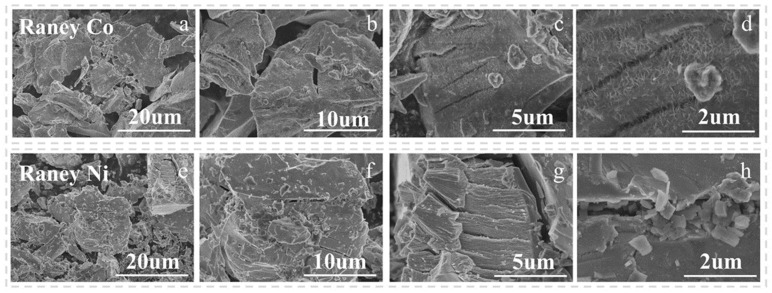
SEM images of Raney Co (**a**–**d**) and Raney Ni (**e**–**h**) at different scale sizes.

Moreover, the difference in surface morphology between Raney Co and Raney Ni may also contribute to their different catalytic performances. The SEM images of Raney Ni and Raney Co at different scale sizes are shown in Figure 5. In Figure 5a,b,e,f, there is no significant difference in the morphology of these two catalysts. With the increase in size (in Figure 5c,d), it can be observed that the Raney Co surface is rougher, with a scale-like structure. And in Figure 5g,h, the Raney Ni presents a smoother surface than Raney Co. The rougher surface of Raney Co with scale-like structures may provide more active sites for the reaction, while the smoother surface of Raney Ni may afford fewer active sites. The surface morphology also affects the diffusion of reactants on the catalyst surface. Raney Co and Raney Ni exhibited pronounced temperature-dependent differences in selectivity toward DCF reduction, governed predominantly by their intrinsic hydrogenation properties. Raney Co demonstrated moderate hydrogenation activity, selectively reducing the nitrile group (C≡N) to a primary amine while preserving the furan ring integrity below 60 °C. In contrast, Raney Ni, owing to its excessively strong hydrogenation capability, tended to induce over-hydrogenation at temperatures above 40 °C, leading to complete saturation of the furan ring C=C bonds and subsequent formation of undesired by-products. Consequently, owing to its moderate hydrogenation activity, Raney Co exhibited better performance during the DCF hydrogenation reaction.

In this work, solvents including EtOH, DMF, PhMe, 1,4-Diox, THF and DCM were screened to demonstrate the critical effect of solvents on the liquid-phase hydrogenation reaction. In a DCM and THF solvent environment, DCF could not be hydrogenated to BAMF (Table 2, entries 1, 2). The solvents 1,4-Diox and PhMe played more of a role in the selectivity of BAMF compared to the former two solvents, however the effects were minimal: only 1.4% and 3.3%, respectively. (Table 2, entries 3, 4). When DMF and EtOH were used as solvents, they provided a favorable solvent environment for the efficient catalytic conversion of DCF to BAMF (Table 2, entries 5, 6). Especially for EtOH, a high selectivity of 87.7% to BAMF was achieved at a DCF conversion of 96.2%. Usually, in a liquid-phase reaction, the higher the solvent’s ability to dissolve H_2_, the more favorable it is to increase the concentration of active hydrogen on the catalyst surface [40,41,42]. The reasons for these solvents’ low conversion rates and low selectivity may be insufficient hydrogen solubility (DCM, 1,4-dioxane, PhMe) or excessively low polarity to stabilize the intermediate (THF). Both EtOH and DMF provide adequate hydrogen solubility with moderate polarity, though EtOH exhibits higher catalyst activity than DMF. Therefore, EtOH was chosen as the optimal solvent for the catalytic hydrogenation of DCF.

**Figure 6:**
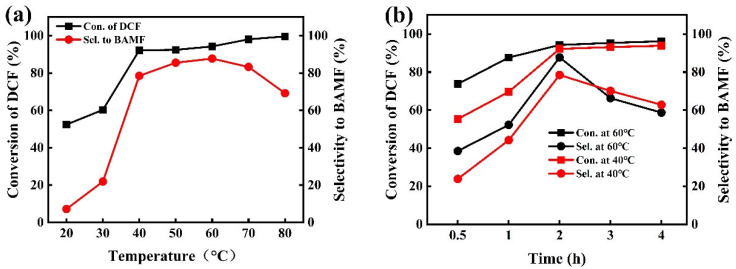
Effects of reaction temperature and reaction time on the hydrogenation of DCF to BAMF: (**a**) effect of reaction temperature at 2 h; (**b**) effect of reaction time at 40 °C and 60 °C. (Reaction conditions: 0.2 g DCF, 2 MPa H_2_, 0.1 g Raney Co, 0.05 g NaOH, 10 mL EtOH).

The effects of reaction temperature and reaction time were studied and are shown in Figure 6. The DCF conversion was positively correlated with the reaction temperature, with a significant increase from 20 °C to 40 °C and a slow increase from 40 °C to 80 °C. The BAMF selectivity showed a volcano curve with the reaction temperature, with a notable rise from 20 °C to 40 °C, followed by a gradual increase from 40 °C to 60 °C, peaking at 60 °C (87.7%), and then slowly declining from 60 °C to 80 °C. As the temperature rises, the catalyst activity increases, leading to a higher hydrogenation rate of DCF and improved conversion efficiency. At elevated temperatures, the DCF conversion rate reaches a plateau phase, which may be attributed to approaching thermodynamic equilibrium, resulting in a slowdown in growth. The effect of reaction time was investigated at 40 °C and 60 °C, respectively. The DCF conversion and BAMF selectivity both increased gradually within the first two hours. The DCF conversion stabilized after 2 h, while there was an obvious decreasing trend of BAMF selectivity. The decreasing selectivity of BAMF was attributed to a deep hydrogenation of BAMF to BATF or another side reaction (Table S3, entries 3–5, 8–10). The specific by-product generation pathways can be found in the subsequent reaction pathway analyses.

Based on the above experimental results, the optimal reaction parameters for DCF to BAMF on Raney Co were determined as follows: EtOH was selected as the solvent, the reaction time was set at 2 h and the reaction temperature was maintained at 60 °C. Under the optimal parameters and increasing the catalyst dosage to 0.4 g, the conversion of DCF reached 99.2%, with the selectivity of BAMF reaching 94.5%.

**Figure 7:**
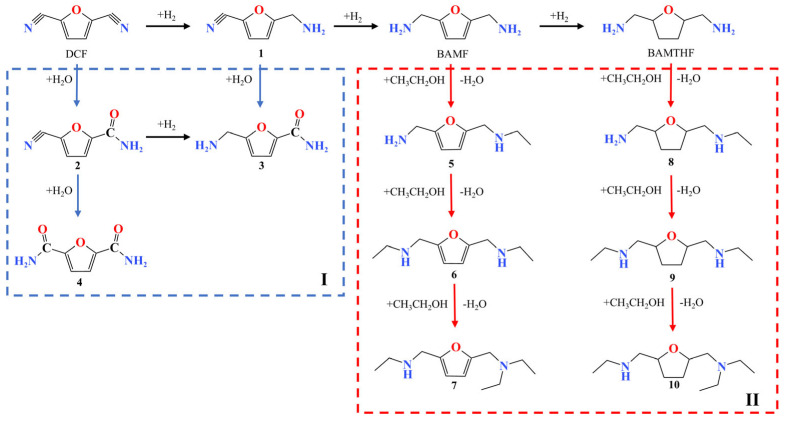
Possible reaction pathways for the production of BAMF and by-products during the catalytic hydrogenation of DCF on Raney Co. (I) Reaction pathway of substrate with water. (II) Reaction pathway of the substrate with ethanol.

To more accurately identify the generated by-products during the DCF hydrogenation process, the products were analyzed using GC-MS. The reaction pathway for the catalytic hydrogenation of DCF in the ethanol system is summarized as presented in Figure 7. The cyano group at one end of DCF reacts with two molecules of H_2_ to form intermediate **1**, then the cyano group at the other end reacts with another two molecules H_2_ to form BAMF. With further hydrogenation, the furan ring of BAMF is hydrogenated to form BATF, which is the main by-product. Along with the primary reaction, several side reactions also occur. There are two pathways for the formation of the by-products: the generation of amides compounds (**2**–**4**) in part I and the generation of secondary and tertiary amines (**5**–**10**) in part II. In part I (shown by the blue arrows), the cyano group of DCF reacts with water to form **2**, and **2** could be hydrogenated to form **3** or further react with water to form **4**. In part II (shown by the red arrows), BAMF reacts with ethanol to form the secondary amine **5**, **5** continues to react with ethanol to form the secondary amine **6**, and then **6** continues to react with ethanol to form the tertiary amine **7**. The reaction pathway between BATF and ethanol is identical to the reaction between BAMF and ethanol, resulting in by-products **8**–**10**. Therefore, in a further industrial experiments, attention should be paid to the side reaction with water or ethanol.

## 3. Experiment

### 3.1. Chemicals

The chemicals of 2,5-Furandicarboxylic acid (98%), SOCl_2_ (thionyl chloride, AR), DMF (N,N-Dimethylformamide, 99%), dichloromethane (AR), dichloroethane (AR), ammonium hydroxide (AR), EtOH (Absolute Ethanol 99.5%) (AR), dioxane (98%), ACN (Acetonitrile, 99%), PhMe (Methylbenzene, 99%), THF (Tetrahydrofuran 99%), DCM (Dichloromethane, 98%), adiponitrile (AR) and H_3_PO_4_ (AR) were purchased from Innochem Co., Ltd., Pyeongtaek, Republic of Korea. Catalysts of Raney Ni (50 μm) and Raney Co (50 μm) were also purchased from Innochem Co., Ltd., Pyeongtaek, Republic of Korea. The chemicals were used as received, without any further purification processing. Catalysts were washed with absolute ethanol and sealed in absolute ethanol before the hydrogenation test.

### 3.2. The Conversion of 2,5-Furandicarboxylic Acid to 2,5-Dicyanofuran

#### 3.2.1. Preparation of 2,5-Dicyanofuran via a Molten Ammoniated Dehydration of 2,5-Furandicarboxylic Acid

In the preliminary exploratory experiments of this study, the FDCA-diammonium salt-diamide-dicyanide reaction pathway was constructed. Specific experimental details are shown in the supporting information, and the reaction schematic is shown in Figure S1. A mixture of adiponitrile, 2,5-furandicarboxylic acid and catalyst H_3_PO_4_ was added to the reaction vessel. After replacing the air in the vessel with nitrogen, the temperature of the reaction liquid was gradually increased to 260 °C and then maintained for a certain period of time under a certain amount of ammonia gas flow. After the reaction, the product was dissolved and diluted in dioxane and analyzed using high performance liquid chromatography (HPLC).

#### 3.2.2. Preparation of 2,5-Dicyanofuran via a Moderate Ammoniated Dehydration of 2,5-Furandicarboxylic Acid

To avoid the molten process under high temperatures, the FDCA-acyl chloride-amide-nitrile reaction pathway was constructed in this work. Specific experimental details are given in the supporting information. 2,5-Furandicarboxylic acid chloride was obtained by heating and refluxing a solution of N,N-dimethylformamide containing FDCA with SOCl_2_. Next, 2,5-furandicarboxylic acid chloride was added to ammonia to obtain 2,5-furandicarboxamide (FDAM). Finally, FDAM was subjected to a dehydration reaction with SOCl_2_ in N,N-dimethylformamide solution to obtain DCF.

### 3.3. The Hydrogenation of 2,5-Dicyanofuran

The catalytic hydrogenation of DCF was carried out in a 25 mL stainless-steel reactor. Prior to the reaction, a certain amount of DCF, solvent, catalyst and NaOH were added into the reactor. The reactor was purged with N_2_ five times to replace the residual air. The reactor was purged with H_2_ five times to replace N_2_, and finally charged with H_2_ to 2 MPa. The reaction was carried under a stirring speed of 1000 rpm. After the reaction, the liquid product was separated from the catalyst and subjected to gas chromatography-mass spectrometry for analysis.

### 3.4. The Analysis of Products

HPLC: High performance liquid chromatography (HPLC Agilent 1260, Agilent Technologies, Santa Clara, CA, USA) was used for quantitative analysis of the products, equipped with a Spursil C18 column and a Agilent 1260 Infinity II DAD WR detector(Agilent Technologies, Santa Clara, CA, USA). A mixture of acetonitrile and water with a mass ratio of 1:2 was used as the mobile phase (1 mL/min). The detection wavelength of the UV-vis detector was 254 nm.

GC-MS: The reaction products were quantitatively identified using gas chromatography-mass spectrometry (Thermo-TRACE1300 series GC instrument, Thermo Fisher Scientific, Waltham, MA, USA) equipped with a DB-17 column (60 m × 0.25 mm × 0.25 μm) and a flame ionization detector (FID) operating at 300 °C. The carrier gas was N_2_ with a flow rate of 1.0 mL min^−1^. A Fourier transform ion cyclotron resonance mass spectrometer (FT-ICR MS, Thermo Fisher Scientific, Waltham, MA, USA) with a magnetic field intensity of 15 T in the positive ion mode (ESI^+^) was used for testing.

Calculation of product composition: The conversion (Y) and product selectivity (X) of DCF were calculated using the area normalization method using the following equations.

$$Y=1-\frac{A_{1}f_{1}}{\sum_{1}^{n}A_{i}{\times f}_{i}}\times 100\%$$

$$X=\frac{A_{2}f_{2}}{\sum_{1}^{n}A_{i}{\times f}_{i}}\times 100\%$$

Y—Conversion of HMF

X—Selectivity of BAMF

A_1_—The peak area of HMF

A_2_—The peak area of the product BAMF

A_i_—The peak area of i

f_1_—The relative molar correction factor for HMF

f_2_—The relative molar correction factor of BAMF

f_i_—The relative molar correction factor of i

### 3.5. The Characterization Method

TG-MS (thermogravimetric and mass spectrometry analysis): TG-MS was performed on a (NETZSCH) STA449F5-QMS403D(NETZSCH Scientific Instruments, Gebrüder-Netzsch-Straße, Germany) with a heating rate of 10 °C/min from 30 °C to 600 °C.

SEM (Scanning electron microscopy): SEM was used to analyze the surface morphology of Raney Co and Raney Ni on a Hitachi S4800(Hitachi High-Tech Corporation, Tokyo, Japan) with an accelerating voltage of 5.0 kV. Before the sample was observed, a small powder sample was glued to a conductive tape and sprayed with Pt for 90 s to achieve ion splitting.

## 4. Conclusions

A novel reaction pathway enabling the transformation of bio-based FDCA to the polyamide monomer BAMF has been developed. The present study is the first to confirm that FDCA is thermally unstable above 200 °C and that a molten ammoniated dehydration route used for aliphatic diacids is not suitable for bio-based 2,5-furandicarboxylic acids. This is because the high temperature required for ammonium salt dehydration contradicts the thermal stability of FDCA. Building on this finding, the present study developed the acyl chloride-amide-nitrile route suitable for bio-based FDCA with very mild reaction conditions, followed by catalytic hydrogenation over heterogeneous catalysts for the preparation of BAMF. SOCl_2_ activates the stable carboxyl group and then acts as a dehydrating agent to efficiently dehydrate the intermediate FDAM to obtain DCF. In the following hydrogenation reaction, Raney Co showed excellent catalytic hydrogenation performance and achieved a 94.5% yield of BAMF. Calculated from FDCA, a 60.5% yield of BAMF was achieved. Guided by industrial applicability considerations, this study achieved the first exploration of bio-based FDCA conversion to BAMF, which provides the theoretical basis and reference experience for the rational preparation of bio-based diamine monomers.

## Figures and Tables

**Table 1:** The results of the FDAM dehydration reaction.

Entry	SOCl_2_/FDAM (Equiv.)	DMF/FDAM (Equiv.)	Time (h)	Temperature (°C)	Yield of DCF (%)
1 ^a^	6	1.6	2	78	0.0
2 ^a^	12	1.6	3	78	0.1
3	17	0	7	−5	0.0
4	4.25	21.5	7	−5	43.6
5	34	43	7	−5	21.0
6	8.5	43	7	−5	42.9
7	4.25	43	7	−5	64.0
8	2.12	43	7	−5	45.8
9	4.25	43	3	−5	59.2
10	4.25	43	6	−5	60.3
11	4.25	43	10	−5	56.8
12	4.25	43	25	−5	57.0
13	4.25	43	32	−5	57.1
14	4.25	43	49	−5	55.3
15	4.25	43	55	−5	54.0
16	4.25	43	75	−5	54.2

^a^ using dichloroethane as solvent.

**Table 2:** The catalytic performance of DCF to BAMF in different solvents.

Entry	Solvent	H_2_ solubility Constant (10^−5^ g/g)	Polarity	Conversion of DCF (%)	Selectivity to BAMF (%)
1	DCM	2.14	3.4	2.6	-
2	THF	3.02	4.2	13.4	-
3	1,4-Diox	2.04	4.8	15.2	1.4
4	PhMe	2.46	2.4	56.9	3.3
5	DMF	2.51	6.4	85.2	67.6
6	EtOH	3.16	4.3	96.2	87.7

(Reaction conditions: 0.2 g DCF, 2 MPa H_2_, 60 °C, 2 h, 0.1 g Raney Co, 0.05 g NaOH, Solvent = 10 mL).

## Data Availability

The original contributions presented in this study are included in the article/Supplementary Material. Further inquiries can be directed to the corresponding author(s).

## Supplementary Materials

The following supporting information can be downloaded at https://www.mdpi.com/article/10.3390/molecules30224336/s1. Figure S1. The experiment setup of molten ammoniated dehydration of FDCA; Figure S2. The HPLC chromatogram for the products (collected in separator) of molten ammoniated dehydration of FDCA; Figure S3. The TG-MS results of FDCA under N_2_ atmosphere; Figure S4. The products under different temperature of FDCA molten ammoniated dehydration at various temperature; Figure S5. The product of FDAM dehydration reaction under 78 °C; Figure S6. (a) ^1^H NMR and (b) ^13^C NMR (DMF-d7) of 2,5-Furandicarboxamide; Figure S7. Mass spectrum of 2,5-furandicarboxamide; Figure S8. (a) ^1^H NMR and (b) ^13^C NMR (CD_2_Cl_2_-d2) of 2,5-furandicarboxylic acid chloride; Figure S9. Mass spectrum of 2,5-Furandicarboxylic diethyl ester; Figure S10. N_2_ adsorption–desorption isotherm (a) and pore diameter (b) of Raney Co and Raney Ni; Figure S11. Mass spectrum of by-products; Table S1. Structural characteristics of the Raney Co and Raney Ni; Table S2. Comparison of hydrogenation effect of DCF from 20 °C to 80 °C under different catalysts; Table S3. Effect of catalyst dosage on the catalytic hydrogenation of DCF over Raney Co.

## Abbreviations

HMF	5-Hydroxymethylfurfural;
FDCA	2,5-Furandicarboxylic acid;
HMDA	Hexamethylenediamine;
FDCC	2,5-furandicarboxylic acid chloride;
HMFA	5-Hydroxymethylfurfurylamine;
FDAM	2,5-Furandicarboxamide
DCF	2,5-Dicyanofuran;
BAMF	2,5-Bis(aminomethyl)furan;
BATF	2,5-Bis-(aminomethyl)tetrahydrofuran;
