# Supplementary material for: A Potential Pathway for the Synthesis of Biomass-Based Polyamide Monomer 2,5-Bis(aminomethyl)furan from 2,5-Furandicarboxylic Acid
Source: Molecules. 2025 Nov 8;30(22):4336. doi: 10.3390/molecules30224336 (PMC12654685; doi:10.3390/molecules30224336)
Supplement: Supplementary file 1 [file molecules-30-04336-s001.zip › molecules-3934721-supplementary.pdf]

Supporting Information

A Potential Pathway for the Synthesis of Biomass-Based Polyamide Monomer 2,5-Bis(aminomethyl)furan from 2,5-Furandicarboxylic Acid

Cong Wang †, Xin Li *,†, Junqi Zhao *, Bin Sun, Enquan Wang, Xuhong Mu * and Xiaoxin Zhang

State Key Laboratory of Petroleum Molecular & Process Engineering, SINOPEC Research Institute of Petroleum Processing, Beijing 100083, China; wangcong.ripp@sinopec.com (C.W.)

* Correspondence: lixin.ripp@sinopec.com (X.L.); zhaojq.ripp@sinopec.com (J.Z.); muxuhong.ripp@sinopec.com (X.M.); Tel.: +86-010-82368823 (X.L.)

† These authors contributed equally to this work.

Supplementary Figures and Tables

Figure S1. The experiment setup of molten ammoniated dehydration of FDCA.

Figure S2. The HPLC chromatogram for the products (collected in separator) of molten ammoniated dehydration of FDCA.

Figure S3. The TG-MS results of FDCA under N_2 atmosphere.

Figure S4. The products under different temperature of FDCA molten ammoniated dehydration at various temperature.

Figure S5. The product of FDAM dehydration reaction under 78 °C.

Figure S6. (a) ^{1}H NMR and (b) ^{13}C NMR (DMF-d7) of 2,5-Furandicarboxamide

Figure S7. Mass spectrum of 2,5-furandicarboxamide.

Figure S8. (a) ^{1}H NMR and (b) ^{13}C NMR (CD_2Cl_2-d2) of 2,5-furandicarboxylic acid chloride.

Figure S9. Mass spectrum of 2,5-Furandicarboxylic diethyl ester.

Figure S10. N_2 adsorption-desorption isotherm(a) and Pore diameter(b) of Raney Co and Raney Ni.

Figure S11. Mass spectrum of by-products.

Table S1. Structural characteristics of the Raney Co and Raney Ni.

Table S2. Comparison of hydrogenation effect of DCF from 20 °C to 80°C under different catalysts.

Table S3. Effect of catalyst dosage on the catalytic hydrogenation of DCF over Raney Co.

1. The test of FDCA to DCF via molten ammoniated dehydration through ammonium salt-amide-nitrile.

The reaction flow diagram for molten ammoniated dehydration is shown in Figure S1. Prior to test, the mixture of 2 g of FDCA, 10 g of adiponitrile (as solvent) and 0.2193 g of H_3PO_4 (as catalyst) were added in a stirred-tank reactor. Subsequently, the system was charged with nitrogen to force out the air, and gradually heated up to 260 °C in the nitrogen atmosphere. The NH_3 flow was charged into the reactor through a heated stainless steel pipe. The products were condensed by the condenser and collected in the separator. The final exhaust was treated by a tail gas absorption device. After reaction, the reaction liquid were solved in dioxane and analyzed by high performance liquid chromatography.

2. The test of FDCA to DCF via moderate ammoniated dehydration through acyl chloride-amide-nitrile.

7.8047 g (0.05 mol) of FDCA and 20.8198 g (0.175 mol) of $SOCl_2$ were added into a 100 mL three-necked flask. After stirring well, 0.0365 g (41 μL) of N,N-dimethylformamide was added, and the molar ratio of N,N-dimethylformamide to FDCA was 0.01:1. The flask was ramped up to reflux at a temperature of about 78-87 °C. And the reaction was carried out for about 2.0 h under the reflux condition until the reaction solution was a yellowish-green transparent liquid. At the end of the reaction, the excess $SOCl_2$ was distilled under reduced pressure at the original reflux temperature. The remaining solid was recrystallized twice with hexane and dried at 110 °C for 12 h. White needle-like crystals of 2,5-furandicarboxylic acid chloride were obtained.

In a 100 mL three-necked flask, 15 mL of dichloromethane and 17 mL (about 0.2264 mol~0.2511 mol) of ammonia (25 wt.%~28 wt.%) were added and mixed homogeneously under an ice-salt bath at -15 °C. And 8.2311 g (0.0427 mol) of 2,5-furandicarbonyl chloride and 15 mL of dichloromethane were mixed homogeneously and added dropwise to the above -15 °C mixture of ammonia and dichloromethane. The molar ratio of the ammonia source to 2,5-furandicarbonyl chloride was about 5.30~5.89:1 and the temperature was maintained at -15 °C until the addition was

completed. And then the temperature of the flask was maintained at 30 °C in a water bath for 2 hours. At the end of the reaction, excess ammonia was evaporated by distillation under reduced pressure. The remaining white solid was washed with deionized water, filtered, and dried at 100 °C for 12 h. The white solid 2,5-furandicarboxamide was obtained.

In a 10 mL two-necked flask, 4.994 mL (43 equiv.) of N,N-dimethylformamide and 0.4630 g (4.25 equiv.) of $SOCl_2$ were added with the temperature controlled at -5 °C. 0.2312 g (0.0015 mol) of diamide was added slowly. Under a temperature of of -5 °C, the reaction was carried out for 6 h. At the end of the reaction, the reaction solution was quenched by adding ice water into the flask. The residual $SOCl_2$ was evaporated under reduced pressure. The reaction product was analyzed by high performance liquid chromatography.

3. Supplementary Figures and Tables

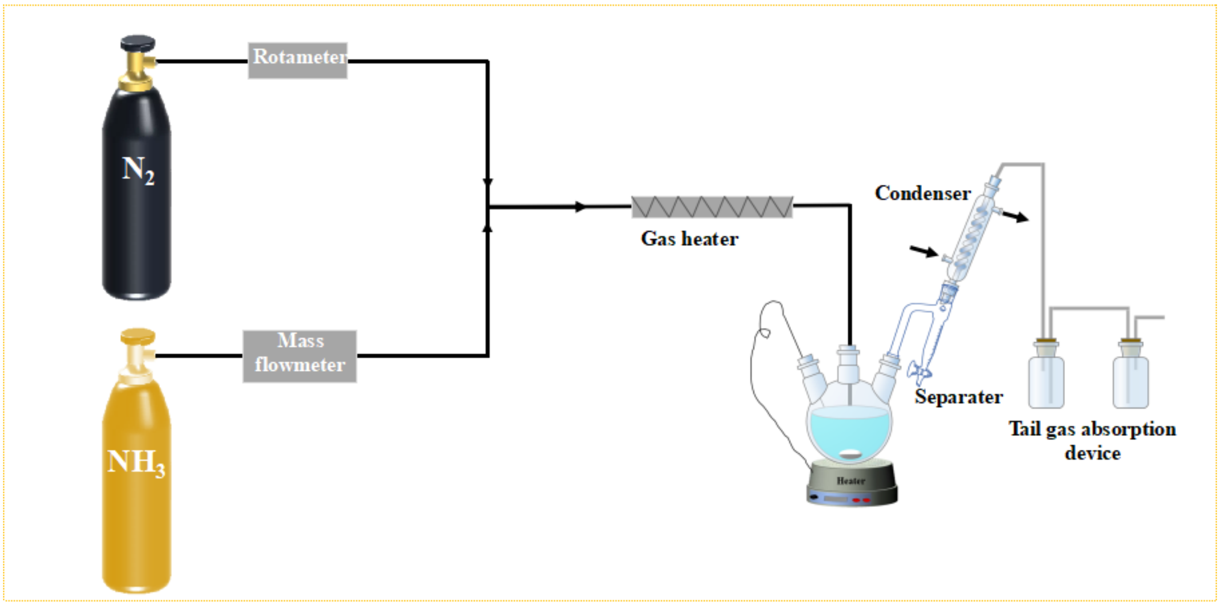

Figure S1. The experiment setup of molten ammoniated dehydration of FDCA.

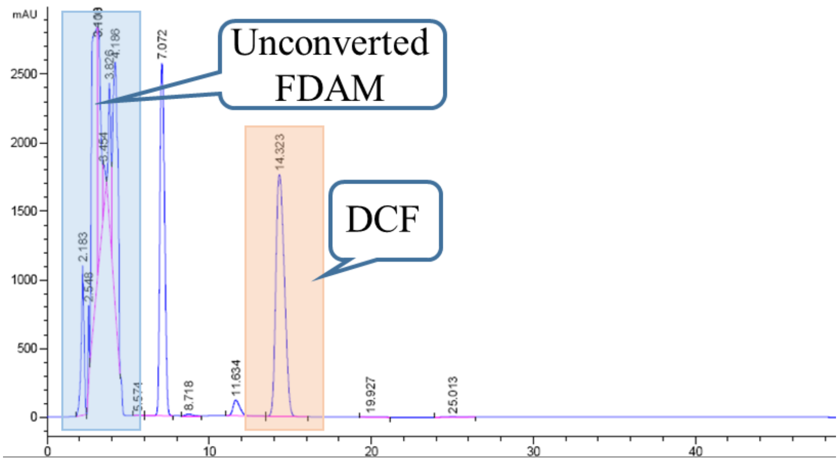

Figure S2. The HPLC chromatogram for the products (collected in separator) of molten ammoniated dehydration of FDCA.

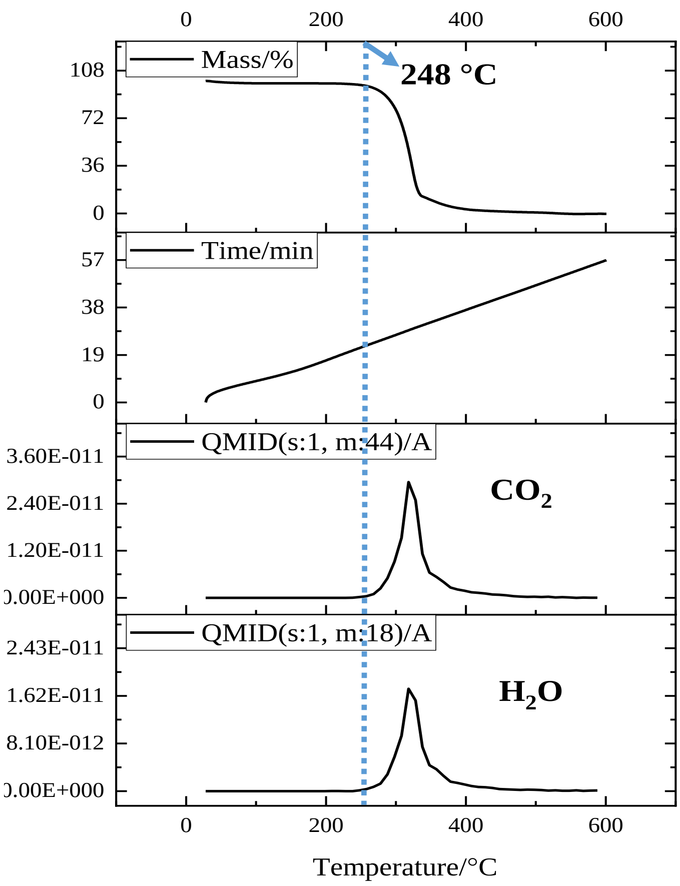

Figure S3. The TG-MS results of FDCA under N_2 atmosphere.

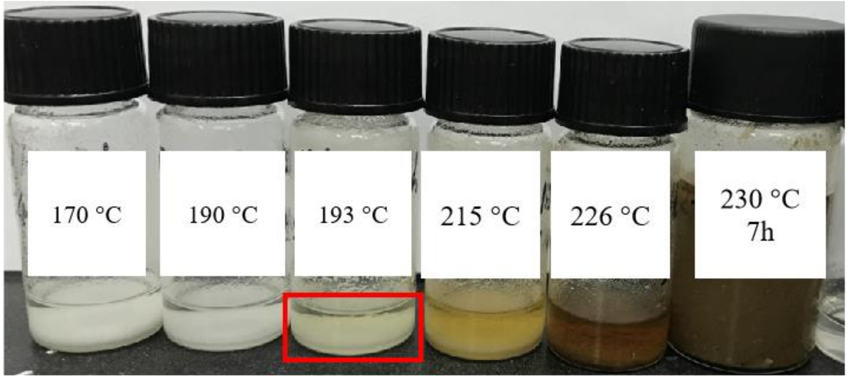

Figure S4. The products under different temperature of FDCA molten ammoniated dehydration at various temperature.

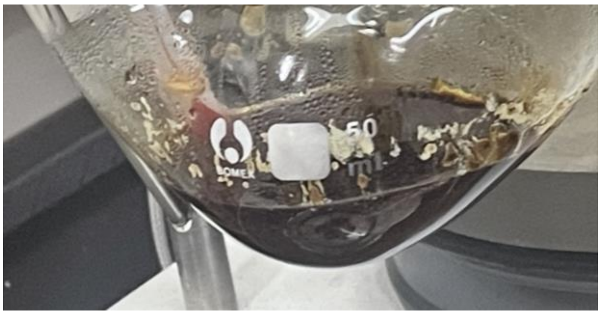

Figure S5. The product of FDAM dehydration reaction under 78 °C.

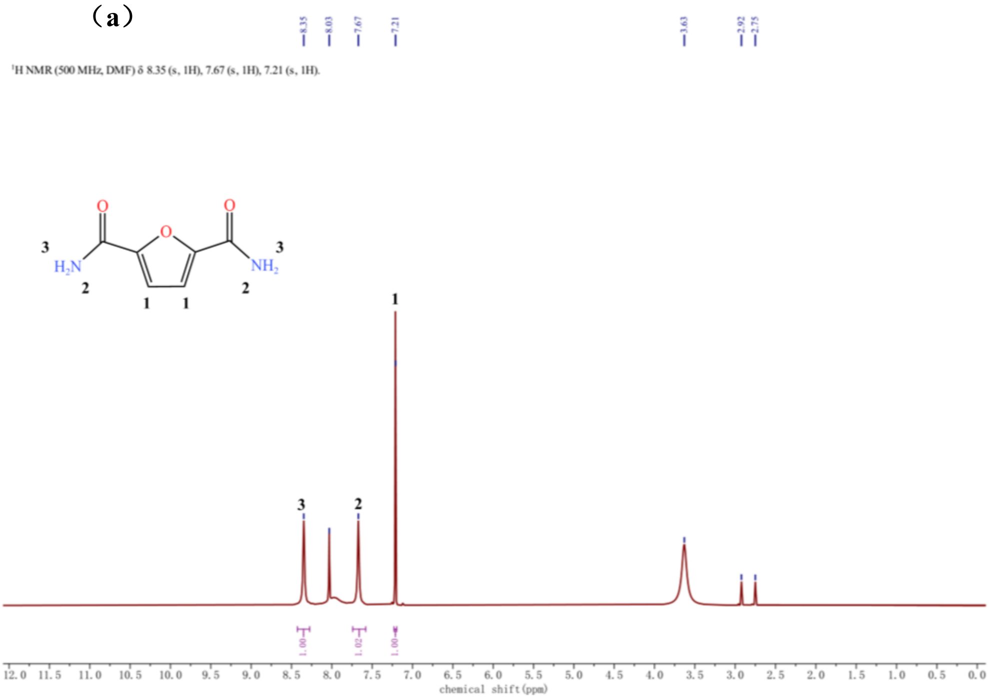

(b)

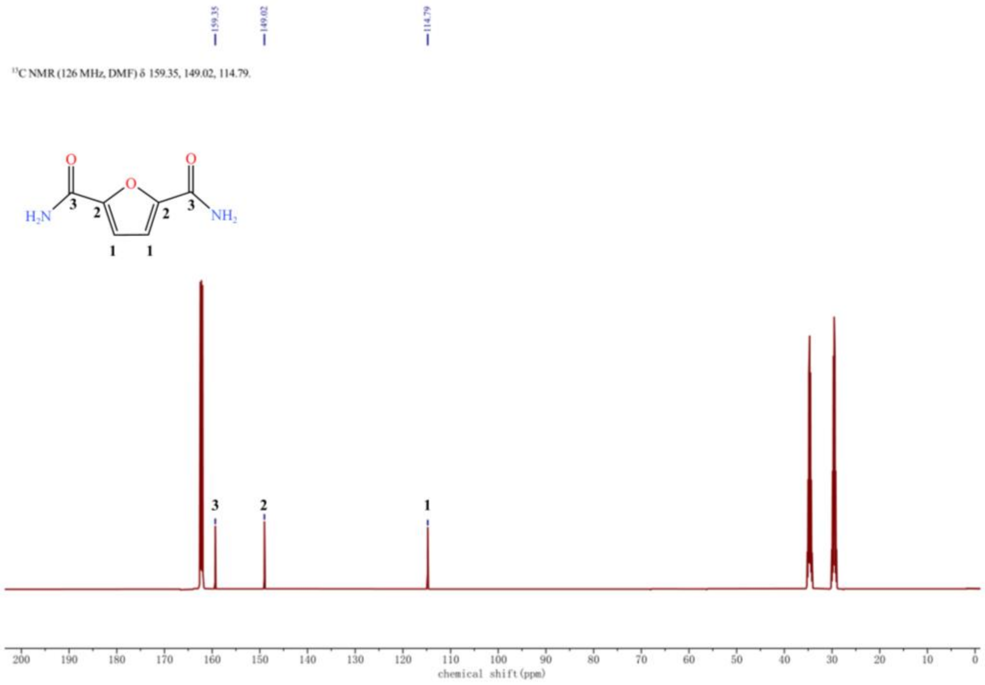

Figure S6. (a) ^{1}H NMR and (b) ^{13}C NMR (DMF-d7) of 2,5-Furandicarboxamide.

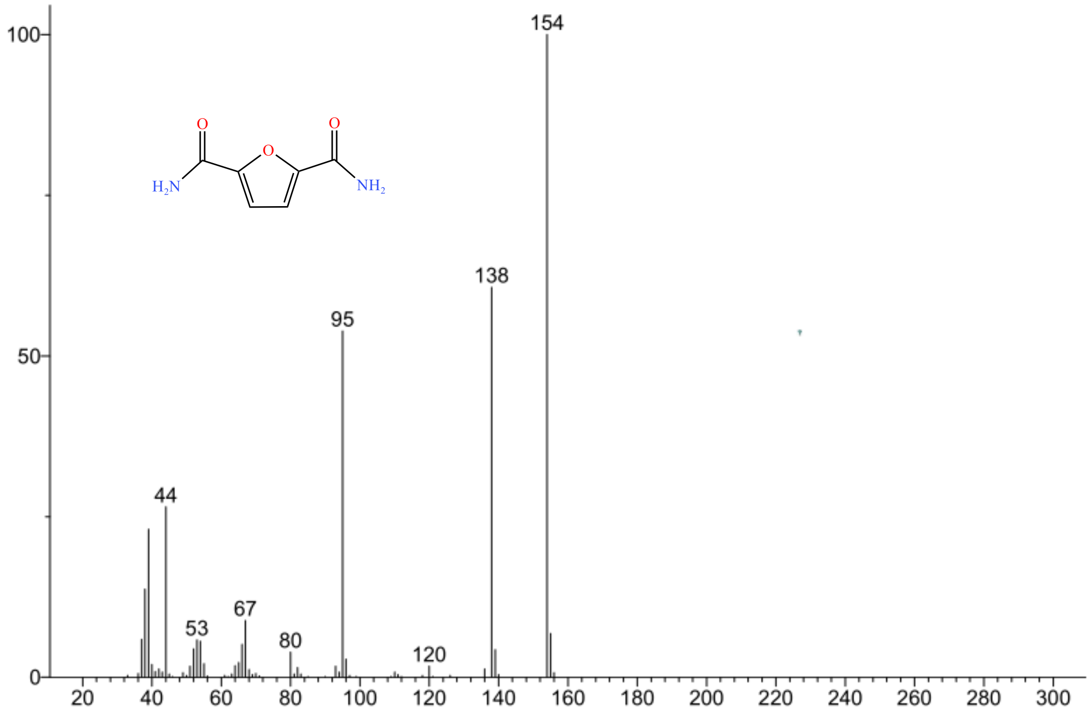

Figure S7. Mass spectrum of 2,5-furandicarboxamide.

(a)

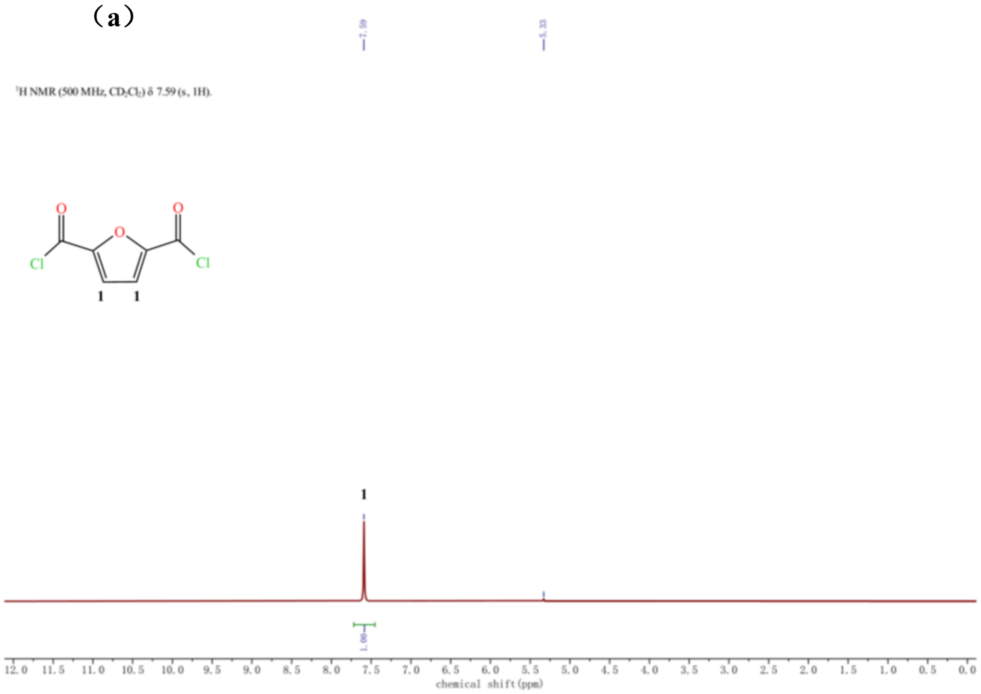
7.59
5.33
1H NMR (500 MHz, CD2Cl2) δ 7.59 (s, 1H).
O
Cl
1
1.00
12.0 11.5 11.0 10.5 10.0 9.5 9.0 8.5 8.0 7.5 7.0 6.5 6.0 5.5 5.0 4.5 4.0 3.5 3.0 2.5 2.0 1.5 1.0 0.5 0.0
chemical shift (ppm)

(b)

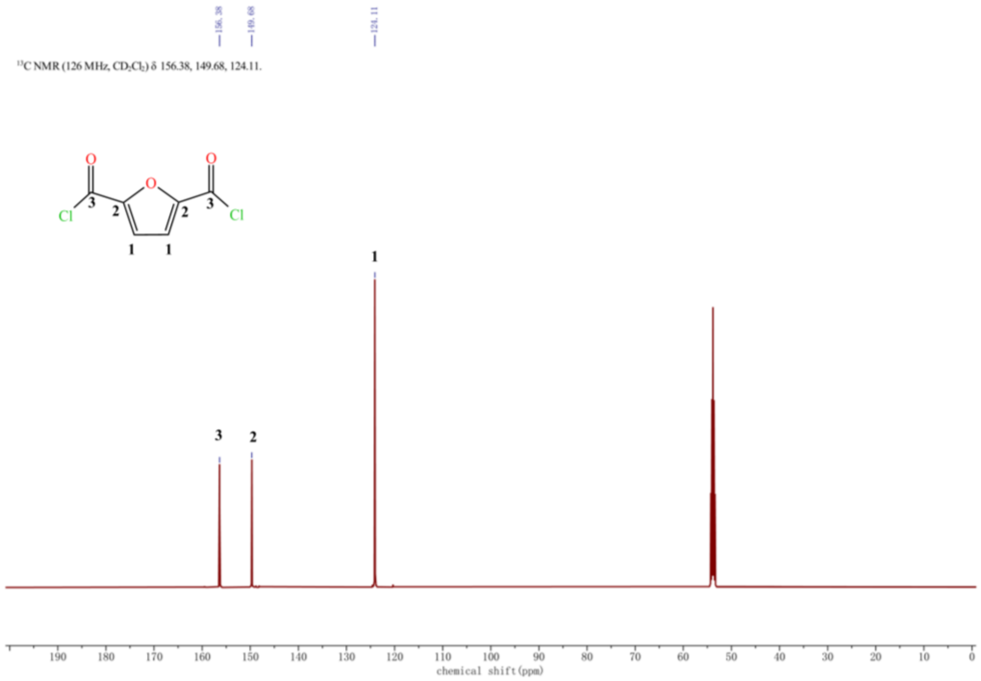
156.38
149.68
124.11
13C NMR (126 MHz, CD2Cl2) δ 156.38, 149.68, 124.11.
O
Cl
1
2
3
190 180 170 160 150 140 130 120 110 100 90 80 70 60 50 40 30 20 10 0
chemical shift (ppm)

Figure S8. (a) ^{1}H NMR and (b) ^{13}C NMR (CD_2Cl_2-d2) of 2,5-furandicarboxylic acid chloride.

Owing to the thermal instability of 2,5-furandicarboxylic acid chloride, its presence is difficult to demonstrate directly by mass spectrometry. Therefore, it is reacted with ethanol to form 2,5-furandicarboxylic diethyl ester, thereby providing indirect structural evidence.

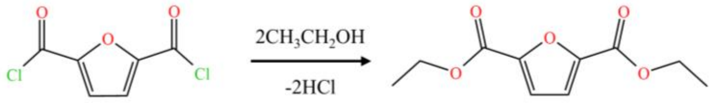

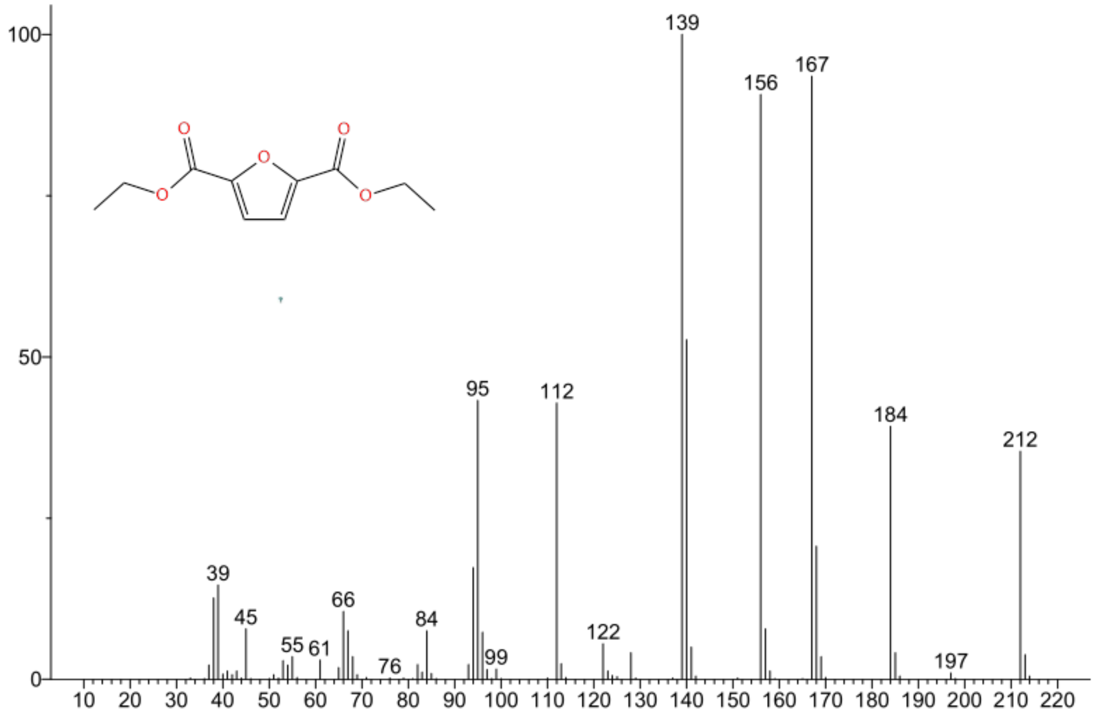

Figure S9. Mass spectrum of 2,5-Furandicarboxylic diethyl ester.

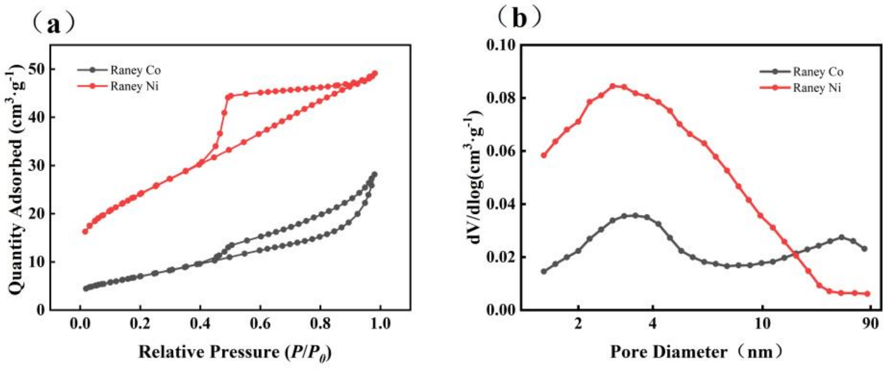

Figure S10. N_2 adsorption-desorption isotherm(a) and Pore diameter(b) of Raney Co and Raney Ni.

Fourier transform ion cyclotron resonance mass spectrometer (FT-ICR MS) with magnetic field intensity of 15T was used for testing. Positive ion mode (ESI+) of electrospray ionization source.

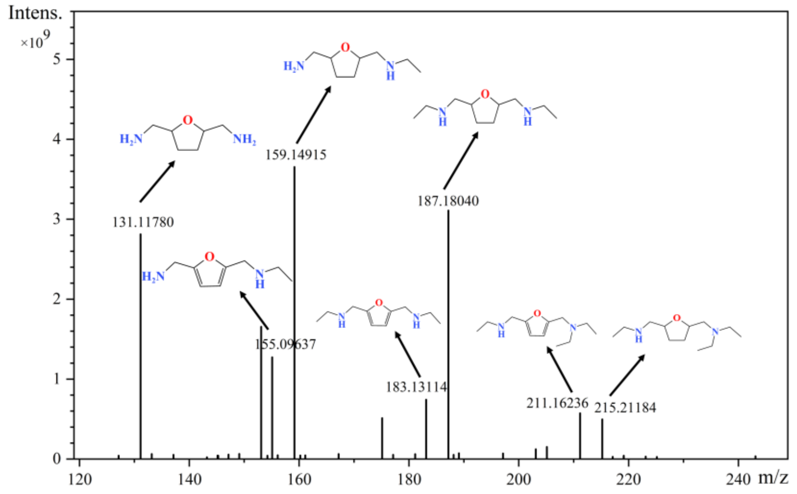

Figure S11. Mass spectrum of by-products.

Table S1. Structural characteristics of the Raney Co and Raney Ni.

Catalyst	SA_{BET} (m^2•g^{-1})	Pore size (nm)	Pore volume (cm^{-3}•g^{-1})
Raney Co	25.48	5.83	0.00092
Raney Ni	86.46	3.60	0.01325

Table S2. Comparison of hydrogenation effect of DCF from 20 °C to 80°C under different catalysts.

Entry	Catalyst	Temperature (°C)	Con. of DCF (%)	Sel. to BAMF (%)	Other (%)
1	Raney Co	20	52.4	7.2	92.8
2	Raney Co	40	92.2	78.5	21.5
3	Raney Co	60	96.2	87.7	12.3
4	Raney Co	80	99.6	69.2	30.8
5	Raney Ni	20	55.0	34.8	65.2
6	Raney Ni	40	85.1	73.5	26.5
7	Raney Ni	60	87.6	57.9	42.1
8	Raney Ni	80	97.2	32.0	68.0

(Reaction conditions: 0.2 g DCF, 2 MPa H_2, 2 h, 0.1 g Raney Co, 0.05 g NaOH, 10 mL EtOH)

Table S3. Effect of catalyst dosage on the catalytic hydrogenation of DCF over Raney Co

Entry	Catalyst	Temperature (°C)	Time (h)	Con. of DCF (%)	Sel. to BAMF (%)	Sel. to BATF (%)	Other (%)
1	Raney Co	60	0.5	73.7	38.5	—	61.5
2	Raney Co	60	1	87.6	52.2	—	47.8
3	Raney Co	60	2	94.2	87.7	1.4	10.9
4	Raney Co	60	3	95.4	66.3	8.4	25.3
5	Raney Co	60	4	96.2	58.7	12.9	28.4
6	Raney Co	40	0.5	55.4	23.8	—	76.2
7	Raney Co	40	1	69.6	44.2	—	55.8
8	Raney Co	40	2	92.2	78.5	1.2	20.3
9	Raney Co	40	3	93.1	70.1	4.9	25.0
10	Raney Co	40	4	93.9	62.8	7.6	29.6

(Reaction conditions: 0.2 g DCF, 2 MPa H_2, 60°C, 2 h, 0.05 g NaOH, 10 mL EtOH)
